# “For Me, the Anorexia is Just a Symptom, and the Cause is the Autism”: Investigating Restrictive Eating Disorders in Autistic Women

**DOI:** 10.1007/s10803-020-04479-3

**Published:** 2020-04-09

**Authors:** Janina Brede, Charli Babb, Catherine Jones, Mair Elliott, Cathy Zanker, Kate Tchanturia, Lucy Serpell, John Fox, Will Mandy

**Affiliations:** 1 Research Department of Clinical, Educational and Health Psychology, University College London, 1-19 Torrington Place, London, WC1E 6BT UK; 2 School of Psychology, Cardiff University, Park Place, Cardiff, CF10 3AT UK; 3 Department of Psychological Medicine, Institute of Psychiatry, Psychology & Neuroscience, King’s College London, De Crespigny Park, London, SE5 8AF UK; 4 Haverfordwest, Wales, UK; 5 Leeds, England, UK

**Keywords:** Autism, Females, Anorexia nervosa, Eating disorders, Co-occurrence, Qualitative research

## Abstract

Autistic women are overrepresented among people in treatment for Anorexia Nervosa (AN). The current study aimed to: (1) better understand how AN develops and persists in autistic individuals from the perspective of autistic women, parents and healthcare professionals; (2) derive a theoretical model of restrictive eating difficulties in autism. We conducted 44 semi-structured interviews and used Thematic Analysis to identify patterns of meaning across the data. Themes related to sensory sensitivities, social interaction and relationships, sense of self and identity, difficulties with emotions, thinking styles, and a need for control and predictability. We developed a model of potential autism-specific mechanisms underlying restrictive eating difficulties. This study generated novel insights, which have the potential to inform treatment adaptations following empirical testing.

**Electronic supplementary material:**

The online version of this article (10.1007/s10803-020-04479-3) contains supplementary material, which is available to authorized users.

The link between Autism and Anorexia Nervosa (AN) was first suggested in the scientific literature by Christopher Gillberg in 1983, who observed in his clinical work that the two conditions co-occurred within the same families (Gillberg 1983). Since then there has been an increasing interest in the overlap between autism and eating disorders (EDs), particularly AN, with research being conducted mainly in western Europe, specifically in Sweden, the UK and Italy (e.g. Nielsen et al. 2015; Vagni et al. 2016; Westwood et al. 2018). Autism is a lifelong neurodevelopmental condition characterised by difficulties with social interaction and communication, presence of restricted and repetitive behaviours and interests, and differences in sensory processing (American Psychological Association 2013). Autistic individuals frequently experience additional difficulties. Seventy percent of autistic young people present with at least one co-occurring disorder, and 41% have multiple comorbidities (Simonoff et al. 2008). Rates of mental health difficulties are similarly high in autistic adults (Lever and Geurts 2016), and having a co-occurring mental health condition has been identified as a significant predictor of poor quality of life in this population (Mason et al. 2018). A better understanding and treatment of co-occurring mental health difficulties in autistic individuals is therefore vital for enabling them to live happier and healthier lives.

AN is an ED characterised by: (1) a range of restrictive eating behaviours and/or compensatory behaviours resulting in significantly low body weight; (2) an intense fear of weight gain or persistent behaviour that interferes with weight gain; and (3) the undue influence of weight and shape concerns on self-evaluation and behaviour or persistent lack or recognition of seriousness of low body weight (American Psychological Association 2013).

Autistic women have an elevated risk of developing AN, as indicated by the fact that they are substantially overrepresented among people in treatment for AN. Studies have consistently shown that 20–35% of women with AN meet criteria for autism (for review, see Westwood and Tchanturia 2017). In contrast, less than 1% of the general population of women meet criteria for autism (Loomes et al. 2017). Some have argued that the effect of starvation in AN may mimic autistic traits, resulting in a pseudo-autistic presentation that includes temporary cognitive rigidity and poor mentalising ability, which may no longer be present once affected individuals have recovered (Treasure 2013; Hiller and Pellicano 2013). However, there is evidence that the high levels of social and flexibility difficulties observed amongst women with AN cannot simply be understood as a starvation-induced autism phenocopy being mislabelled as ‘true autism’. First, autism prevalence rates remain high even in samples of women who have recovered from AN and are no longer starved (Bentz et al. 2017; Nazar et al. 2018). Second, when rigorous, gold-standard assessment instruments are used, around a quarter of women with AN continue to meet autism diagnostic criteria (Westwood et al. 2017b). Third, studies using retrospective self- or parent-report of autistic behaviours suggest that for many women with AN high autistic traits were already present in childhood and predated their ED (Vagni et al. 2016; Westwood et al. 2018).

Despite a significant proportion of autistic women in ED services, current service provision does not acknowledge or address their needs (Kinnaird et al. 2017,2019b). Evidence from both the UK and Sweden suggests that women with high autistic traits benefit less from current interventions and care pathways and have worse outcomes than other women with AN, experiencing especially low recovery rates and levels of functioning (Nielsen et al. 2015; Tchanturia et al. 2017,2016; Nazar et al. 2018). Qualitative interviews of clinicians working in ED services in the UK found that adaptations to treatment tended to be idiosyncratic and based on the previous experience of individual clinicians, rather than representing a system-wide approach (Kinnaird et al. 2017). Whilst most ED clinicians recognised the importance of considering autism in AN treatment, many did not feel they had enough knowledge to provide adequate treatment for this client group (Kinnaird et al. 2017). In line with this finding, autistic women in treatment for AN in the UK reported that they experience unique needs associated with their autism, which they feel are not met by currently offered treatments (Kinnaird et al. 2019b). Although there will be some differences in how ED services are organised across different countries, given the lack of knowledge on the overlap between the two conditions and lack of official guidance on how to recognise and support autistic women in ED services, these findings are likely to also apply to the service experience of women in other countries. Overall, there is a limited evidence base to guide service improvements for autistic women seeking support for AN (Huke et al. 2013; Westwood and Tchanturia 2017). A first step towards guidance for services would be to develop a testable theoretical model of the specific autism-related processes that might give rise to and maintain restrictive eating behaviours, underlying AN, in autistic individuals.

Within the ED literature, a considerable body of research has established the presence of certain characteristics in AN populations, which are also recognised in autism, and thus are of potential relevance to building models of AN in autistic individuals. These include, but are not limited to, atypical social cognition (e.g. Zucker et al. 2007), difficulties processing emotions (e.g. Lang et al. 2016), weak central coherence (e.g. Oldershaw et al. 2011), and cognitive rigidity (e.g. Westwood et al. 2017a). However, only a few studies have assessed whether such traits are actually associated with autism and/or autistic traits within ED populations. Tchanturia et al. (2013) explored associations between self-reported autistic traits and clinical ED symptoms in 66 individuals with AN and 66 healthy controls. The AN group reported more autistic traits than controls. Autistic traits discriminated between groups related to global thinking, inflexibility of thinking and problems with social interactions, but were not associated with ED symptoms. This suggests that autistic traits may exacerbate factors that maintain the eating disorder rather than cause the eating disorder directly. Lang et al. (2016) found that reduced positive emotion expression was associated with autistic traits and a number of other clinical variables in 66 individuals with AN. Westwood et al. (2017a) investigated the relationship between autistic traits and neuropsychological performance in 99 females with AN. Their results suggest that the presence of autistic traits is related to increased cognitive rigidity in females with AN. Within the autism literature, evidence suggests that sensory sensitivities may play a role in the development of picky eating and food selectivity, both of which are common in autistic individuals (Kuschner et al. 2015; Cermak et al. 2010). However, it is unclear whether sensory sensitivities have a specific impact on the development of EDs, such as AN, in autism (Kinnaird et al. 2019a).

Qualitative interviews with autistic people with AN are important for a better understanding of the autistic features that may contribute to the development and maintenance of their eating difficulties, as this ensures that emerging knowledge is grounded in the lived experience of affected individuals. So far there has only been one relevant study (Kinnaird et al. 2019b), although the study’s main focus was on autistic women’s experience of ED treatment and potential adaptations. Kinnaird et al. (2019b) interviewed nine diagnosed autistic women and four women with high autistic traits about their experience of AN and the treatment they received. Participants reported experiencing their autism and their ED as fundamentally interlinked, with their autistic traits motivating apparent ED behaviours in ways that are not accounted for by traditional models of AN. Participants described how rigidity and inflexibility associated with their autism had contributed towards the development of fixed routines and rituals around food. Participants also felt that commonly assumed motivations, such as a desire to lose weight, low self-esteem, and body image issues, were less relevant in the development of their illness compared to other less typical motivations, such as need for control, sensory difficulties, social confusion, organisational problems surrounding cooking and food shopping, exercise as a method of stimulation, and the ED acting as a special interest (Kinnaird et al. 2019b).

Kinnaird et al.’s findings (2019b) therefore suggest that there may be autism-specific mechanisms underlying AN in autistic women and that restrictive eating difficulties in autistic women, although being labelled AN, may deviate from traditional AN presentations. However, there is a need to gain further understanding of potential autism-specific mechanisms that underpin these restrictive eating difficulties. Specifically, not only are more targeted, in-depth interviews required to extend understanding of the experiences of autistic women with AN, but an approach that integrates the perspectives of autistic women with the views of those who support them will provide more comprehensive insight. Autistic women’s direct accounts of their own experiences should be central to the development of knowledge about them. Triangulation with the views of other groups, specifically those involved in their care, can further enrich the emerging understanding and give insight into the wider recognition of their autistic perspective (Carter et al. 2014). Developing a model that proposes mechanisms underlying restrictive eating difficulties in autistic individuals more generally, rather than just mapping autistic women’s experience of restrictive eating onto our current understanding of AN, will facilitate a discussion of other potentially autism-specific motivations for restrictive eating beyond those that are commonly associated with AN. In addition, such model has the potential to provide a foundation to guide clinical adaptations and will stimulate future research by generating new hypotheses.

The current study brought together the perspectives of autistic women, parents of autistic women, and healthcare professionals to: (1) better understand how AN develops and persists in autistic individuals and (2) derive the first theoretical model of restrictive eating difficulties in autism.

## Methods

### Design

This study employed a qualitative research design, as this allowed us to deepen our understanding of the phenomenon in question and to generate new hypotheses, rather than testing pre-established hypotheses or predictions (Pistrang and Barker 2012). We generated data using semi-structured interviews with individual participants to give participants the freedom to describe their experience in their own words. Thematic Analysis (Braun and Clarke 2006) was used to identify patterns of meaning across the data. This approach was chosen because of its flexibility, which suited both the aim of capturing the phenomenon of interest, i.e. AN in autistic women, as well as the more theory-generating aim of developing a model (Fereday and Muir-Cochrane 2006). Data were interpreted within an essentialist framework, using an inductive approach, with theme development being driven by the data and grounded in participants' experiences.

### Participants

We recruited participants from the following groups: (1) autistic women; (2) parents of autistic women; and (3) healthcare professionals with relevant experience. This was done via social media, the Discover research network (Autistica 2019), and existing contacts. Based on the teams previous experience of conducting qualitative research with autistic women and individuals with eating disorders and on other guidance (Guest et al. 2016), we aimed to recruit 15 participants for each group. We reflected on our progress throughout data collection and stopped recruiting once we estimated that data saturation had been reached. The final sample included 15 autistic women, 13 parents, and 16 healthcare professionals. Participants were distributed across England, Scotland and Wales.

#### Autistic Women

Autistic women were eligible to participate if they met the following inclusion criteria: (1) above the age of 18 years; (2) clinical diagnosis of an autism spectrum disorder (self-report); (3) past or current experience of AN; and (4) living in the UK. We used the 10-item Autism-Spectrum Quotient (AQ-10; Allison et al. 2012) to confirm their autism status. Two of the 17 women recruited scored below the cut-off, and their interviews were not included in the analysis.

The demographics of the autistic women are provided in Table 1. All autistic women had been in contact with services for their ED and other mental health conditions first, often for years before their autism was recognised. Their ED status was varied at the time of study. Some considered themselves to be currently living with AN, some considered themselves to be recovered, and some considered their condition to be improved, but still struggled with aspects of their ED. Women’s Body Mass Index (BMI) was based on self-reported height and weight. Eight women declined to share this information. At the time of the study, the majority of autistic women were not in full-time employment, several were studying at university level, but some had interrupted their education due to their ED, and some held part-time jobs or voluntary positions.

**Table 1:** Demographics for autistic women and autistic daughters of parents who participated in the study

		Age in years	Age of AN diagnosis (in years)	Age of autism diagnosis (in years)	AQ-10	EDE-QS	BMI^b^
Autistic women (N = 16)	Range	23–58	10–34	14–34	7–10	0–26	15–23
	M (SD)	32.6 (10.32)	17.40 (6.07)	29.40 (11.34)	8.73 (1.1)	11.53 (6.49)	18.28 (3.19)
Daughters of parents (N = 12)^a^	Range	15–31	10–25	9–30			
	M (SD)	24.75 (6.36)	15.50 (4.17)	21.17 (7.15)			

*Notes* AN: Anorexia Nervosa; AQ-10: Autism-Spectrum Quotient (Allison et al. 2012); EDE-QS (Eating Disorders Examination Questionnaire Short (Gideon et al. 2016); BMI: Body Mass Index, calculated on self-reported weight and height

^a^Five parent participants were parents of autistic women in this study

^b^Eight women declined to provide details on their weight and height for their BMI to be calculated

#### Parents

The parent sample included five parents of autistic women who also participated in this study, and eight whose daughters did not participate. Parents were eligible to participate if their daughters met the same inclusion criteria as those applied to autistic women, with the exception that their daughters could be below the age of 18 years. One set of parents (father and mother) were interviewed together; all other parents were mothers and were interviewed individually. One mother’s interview was not included in the final analysis because her daughter was one of the participating autistic women who scored below the autism screening cut-off. The demographics for parents’ daughters (Table 1) were similar to those of the autistic women, although they were a slightly younger sample.

#### Healthcare Professionals

Healthcare professionals were identified through contacts of the research team and via snowball sampling, asking professionals who had participated if they were aware of any colleagues who might be suitable. Healthcare professionals had relevant experience of working with autistic individuals with eating difficulties. We invited professionals from different services across the country, with different professional backgrounds and at different stages of their career, to ensure variation in training and work context. On average, they had worked in autism and/or ED services for 10 years (range 2–23 years). They belonged to a variety of professions, including child and adolescent psychiatry (N = 2), adult psychiatry (N = 3), clinical psychology (N = 6), counselling psychology (N = 1), nursing (N = 1), speech and language therapy (N = 1), dietetics (N = 1), and social work (N = 1).

### Procedure

We consulted two autistic women with experience of AN to advise on the interview schedule and procedure to ensure that participation was comfortable and accessible for autistic women. Both women advised on an early draft of the design and one gave detailed feedback on the interview schedule. Both also provided feedback at different stages of the analytic process.

Participant interviews were conducted face-to-face, via Skype, or over the phone and lasted on average for 1 h 23 min (range 43 min–2 h 26 min) with autistic women, 1 h 27 min (range 43 min–1 h 54 min) with parents, and 52 min (range 20 min–1 h 15 min) with healthcare professionals. Basic demographic information (all three groups) and questionnaires (autistic women only) were collected immediately prior to the interview. Interviews were conducted by one of two non-autistic female PhD students. Participants participated in a one-off interview only and were offered £10 to thank them for taking part. Informed consent was obtained from all individual participants included in the study.

### Materials

Semi-structured interview schedules (Supplementary Material) were developed by the research team and via consultation with the autistic advisors. The interview schedule development was guided by the research question of how AN develops and persists in autistic individuals. We intended for the generated data to also aid a separate investigation concerning autistic women’s ED service experience, which will be reported elsewhere. We initially developed the interview schedule for autistic women, and then adapted it as appropriate for the other two groups. Interviews with autistic women covered their experience of autism, AN, factors that might be underlying the development of their AN, as well as their journey towards an autism diagnosis and their ED service experience. After giving them the opportunity to share their experiences more generally, we asked specific questions about the relevance of potential influencing factors in the development of their AN. These were identified from the existing literature and anecdotal accounts e.g., the role of weight and shape concerns and food-related sensory experiences. Parent interviews included questions about their daughters’ autism and AN, how their daughters’ AN had developed, the relevance of the potential influencing factors, and their daughters’ experience in services. We asked professionals how AN and/or autism tends to present in female clients they are working with, their thoughts on the relationship between both conditions, treatment provision for these women, and their experience of working with autistic women with AN. Participants within each group were asked the same key questions, but further prompts were used flexibly to follow up on points as they emerged.

The 10-item Autism-Spectrum Quotient (AQ-10; Allison et al. 2012) was used to confirm autism status and indicate symptom severity. Scores on the AQ-10 range from 0 to 10, with higher scores indicative of the presence of greater autism symptom severity. Using a cut-off score of six, the 10-item version yielded a sensitivity of 0.88, specificity of 0.9 (Allison et al. 2012). Internal consistency for the autistic women in our sample was low (α = 0.29).

The Eating Disorders Examination Questionnaire Short (EDE-QS; Gideon et al. 2016) was used to measure current ED psychopathology. The EDE-QS is a 12-item, single-factor self-report questionnaire, asking participants to indicate how many days during the last week they have experienced various ED symptoms using a 4-point response scale ranging from “0 days” to “6–7 days”. These response options correspond to scores of 0 through 3, with higher scores indicating more severe ED symptoms (max = 36). In addition, the EDE-QS asks participants for their height and weight for BMI to be calculated. The EDE-QS demonstrates sound psychometric properties and is able to distinguish between individuals with and without clinical EDs (Mdn = 17.5 vs. Mdn = 5.0) (Gideon et al. 2016). Internal consistency for our participants was acceptable (α = 0.77).

### Analysis

All interviews were audio-recorded. Interviews were transcribed verbatim and entered into NVivo (version 12; 2018) for analysis. The full transcripts were used in this study.

We used Thematic Analysis (Braun and Clarke 2006) to identify patterns of meaning across the data. Theme development was guided by the overarching research questions of how AN may develop and be maintained in autistic women.

We adhered to guidelines for good practice in qualitative research (Mays and Pope 2000; Pope et al. 2000) to ensure that interpretations of the data were thorough and consistent. We employed a consensus approach to coding. After familiarisation with the interview transcripts, two researchers (JB and CB) jointly analysed the data to avoid relying on a single analyst driving theme development (Hill et al. 1997). Both researchers analysed all transcripts for each group in the opposite order to each other. At least twice during each group’s analysis, JB and CB reviewed and merged each other’s coding to ensure consistency across transcripts. By analysing the transcripts in reverse order, both researchers brought different insights when discussing theme development at various stages of the analysis. It also balanced the weight of each participant’s perspective, ensuring that all voices contributed to the shaping of themes. The transcripts from the three participant groups were analysed separately, starting with the autistic women’s data, before looking for similarities and differences across the data set. This allowed us to develop a comprehensive understanding of the nuanced variations in the different groups’ perspectives, while keeping the autistic women’s direct experiences central to theme development. The researchers also regularly discussed their progress with the wider research team to generate alternative ways of viewing the data and expand their understanding of the data (Barbour 2001), until a consensus on the best way to represent the data was reached. At two points during the analytic process we consulted with the autistic advisors, who commented on the interpretations made by the researchers. Codes and/or themes were adapted if the research team agreed with their interpretation. This ensured that the findings made sense in the context of the advisors’ lived experience and their understanding of the experiences of others within their community.

Once the themes had been identified, further analysis and discussion within the research team led to the development of a model of restricted eating difficulties in autism, which highlights the relationships between themes and underlying processes. The model was developed separately from the themes and adds another layer of interpretation, which was conducted for a more theory-generating purpose. This process included a wider range of approaches of engaging with the data, such as representing relationships spatially by arranging printed codes and themes on the floor, and visually capturing potential processes in diagrams, which then served as the foundation for the model developed. The model is directly informed by, but goes beyond, the qualitative data and themes, generated by the thematic analysis.

## Results

### Thematic Analysis

We structured our codes around six themes, some of which include further subthemes (see Table 2): “sensory sensitivities”, “social interaction and relationships”, “self and identity”, “difficulties with emotions”, “thinking styles”, and “need for control and predictability”. Overall these themes were endorsed by all participant groups, although some were more clearly expressed by some, as will be outlined below. These themes overlap and influence one another, as highlighted in the subsequent theme presentation.

**Table 2:** Overview of themes from thematic analysis

Main themes	Subthemes
Sensory sensitivities	Sensory overload
	Food-specific sensory sensitivities
	Internal and bodily sensations
Social interaction and relationships	
Self and identity	
Difficulties with emotions	
Thinking styles	Literal thinking
	Intense interests
	Rigid thinking
Need for control and predictability	

#### Sensory Sensitivities

Sensory sensitivities contributed to autistic women’s EDs through general sensory overload, food-specific sensory sensitivities, and discomfort and confusion related to internal and bodily sensations.

##### Sensory Overload

Some women reported having aversive sensory sensitivities related to noise, touch and certain lighting. Parents also noted sensory issues as one of the key ways in which their daughters’ autism affected their day-to-day life and related this to meltdowns their daughters experienced. Healthcare professionals observed that many of their autistic clients struggled with the sensory experience of the treatment environment. These experiences of sensory overload appeared to affect autistic women’s eating behaviour, with some women seemingly using the effect of starvation on their body to numb these sensations.

##### Food-Specific Sensory Sensitivities

Almost all of the women experienced food-specific sensitivities related to food texture, taste, smell, temperature, or the mixing of different foods, which limited the range of foods they would eat.

“I've never eaten a tomato in my life because it’s just hard and it’s squidgy in the middle, it just disgusts me. There's absolutely no way I could eat it.[…] And I'm not keen on lettuces either. The fact it’s all mixed up, which in my head it shouldn’t all be mixed up, so for me a salad is actually a terrifying food!” AW08

This quote illustrates how repulsive certain textures and mixing of foods are for this participant. Several healthcare professionals emphasised that autistic women’s motivation for food restriction often related to sensory properties of food, rather than primarily being based on calorie or fat content, which they saw as distinct from other women with AN.

Some women took extreme measures to avoid anticipated negative sensory experiences, refusing to touch certain types of food or even cutting out whole food groups in response to one negative experience, which parents and healthcare professionals related to their rigid thinking style. These food-specific sensory sensitivities were reported to have been present since early childhood, predating the women’s EDs, and continued to interfere with their eating, even for those who had recovered. Most parents recalled their daughters having difficulties during mealtime or when others around them were eating. This mother’s quotes emphasises the impact this had on her daughter's behaviour and on how she was seen by others:

“If somebody else had a packed lunch that had a strong smell, she wouldn’t just say ‘I don’t like the smell of that’. She would just overreact, and the teacher would think she was just badly behaved and stuff. But for her the smell was just unbearable.” P05

Several parents said they had only realised in hindsight that some of their daughters challenging behaviours during mealtimes might have been due to their sensory sensitivities. In line with this, autistic women reported that, particularly when they were younger, others often misunderstood their responses to these sensory experiences or refused to accommodate their sensory sensitivities, which further exacerbated their difficulties.

Some participants felt that autistic women’s sensory-related restrictive eating had increased the chances of them developing an ED, because their relationship to food had always been difficult. Several participants, particularly healthcare professionals, drew parallels with the presentation of Avoidant/Restrictive Food Intake Disorder (ARFID), a recently introduced ED that is not driven by weight and shape concerns (American Psychological Association 2013).

“I think also there is the crossover with ARFID. Being quite restrictive about the kind of foods they will eat, quite fussy about textures, tastes, how it’s prepared. And that can sometimes tip over into more rigid patterns of eating.” HCP02

A few of the women and parents, who had come across this label, even wondered whether this would have been a more appropriate diagnosis than AN, or whether one had morphed into the other.

##### Internal and Bodily Sensations

Hypersensitivity to sensory stimuli also applied to internal sensations. For some women the internal sensations associated with eating, such as feeling bloated or the sensations of digesting food, were very distressing and they reported restricting their eating to avoid these sensations. Although some parents speculated that this might be the case, this was mainly described by autistic women themselves.

“That feeling of putting on weight… that’s what kind of sends me back into restricting food, because it’s not about ‘oh god my stomach looks really big’, it’s more about ‘I don’t like the sensation of how my stomach feels’.” AW11

In contrast, several other women talked about hyposensitivity to internal sensations, which led to difficulties with interoception, i.e. the ability to sense the internal state of the body. This resulted in difficulty recognising and understanding emotions, as well as a difficulty interpreting eating-related sensations, such as hunger and satiety. Some reported consistently missing meals because they failed to notice they were hungry. Others would overeat without realising and then feel so uncomfortable they would subsequently restrict food. This woman described how her difficulties with interoception could result in both:

“I’m not very good at judging my own emotions or physical sensations. I don’t really fully understand my thirst and hunger responses, or my fullness responses, so that really influences my eating because I can binge or miss meals very, very easily.” AW09

Interoceptive difficulties were viewed as a pathway towards establishing ED behaviours, such as restricting food intake for long periods or entering a cycle of bingeing and restriction. For some women this also meant that they had never been able to regulate their eating routine without relying on external cues, such as the time of day or size of a dish, even before their ED started. This was described as an additional challenge in overcoming their ED and developing a healthy eating routine.

Several healthcare professionals pointed out that this seemed to be unique to the presentation of autistic women with AN.

“Girls without autism do feel hunger, but they are actively working against those feelings of hunger. Some of the girls with autism I’ve spoken to don’t seem to recognise it […] there’s something about their sensory profile that possibly means that they don’t experience hunger in quite the same way.” HCP02

#### Social Interaction and Relationships

All participants talked about autistic women having longstanding difficulties with social interaction and communication, including difficulties in friendships and experience of loneliness, bullying and abuse, which affected their eating.

Difficulties with social interaction made them vulnerable to adverse experiences and left them in a constant state of confusion and exhaustion. Restricting their eating was described as a way to cope with social difficulties and distract from or numb consequent emotions. The following quote exemplifies how the emotional burden of losing a friend lead to this woman immersing herself in ED behaviours:

“I think I was lonely a lot after [my only friend changed school] and that affected it, and I could just get engrossed in food and exercise and just forget about everything else.” AW07

In many cases, autistic women’s social difficulties got worse, or their awareness of them increased, as they reached adolescence, which coincided with the onset of their ED.

Another way in which social difficulties might affect restrictive eating, seems to be avoidance of social settings that happen to involve food. For example, several women described how they initially started restricting their food intake when they skipped lunch in school canteens because they felt overwhelmed by the social or sensory environment, did not have anyone to sit with, or wanted to avoid bullies.

“The moment when I stopped eating at school, was because there was a big canteen, lots of people, lots of social stuff going on, lots of noise.” AW03

#### Self and Identity

Almost all participants talked about autistic women lacking a sense of self, feeling different, and not fitting in as central to the development of their EDs. These feelings caused emotional upset, which they reportedly tried to cope with by immersing themselves in ED behaviours. In addition, for some women dieting or focusing on their appearance was used as a way to fit in with peers, or the ED provided a sense of identity.

Autistic women mainly attributed their feelings of difference and lack of sense of self to not having been able to make sense of their autistic experiences. The following quote illustrates how this woman’s lack of understanding of her autism-related differences affected her self-esteem:

“You constantly feel like you’re failing, you constantly feel different, you think it’s all your fault because you don’t know that there is something different about you.” AW08

None of the women had an autism diagnosis when their eating difficulties emerged, and many participants wondered whether the women would have found it easier to cope if they had known they were autistic. Several parents blamed themselves for not recognising their daughter’s difficulties or for not fighting enough to get the right diagnosis in childhood.

“I wonder, if I’d have picked up on the autism earlier, I might’ve been able to prevent the eating disorder. Or at least stop it getting to that severe point.” P10

For some women, struggles with their sense of self led to them focusing on their weight and shape. In an attempt to make sense of their experience of not fitting in, a few women concluded that the reason must relate to their body and appearance.

For others, being exposed to societal messages about the importance of women being thin resulted in them wanting to change their body weight and shape in order to fit in and connect with peers.

“All her life [my daughter] had been surrounded by women who would talk about dieting, you know, I wished my legs weren’t so fat, all those things. And [my daughter] knew that that’s important, even though she didn’t care what she looks like, but she knew that it’s a thing for normal women, for other women, and she wants to be the same.” P12

In a few cases, anorexia and its values, including the desire to be thin, provided a sense of identity that autistic women had been lacking.

“I have never had much of a sense of self, and I think possibly [AN] then became a little bit like an identity. Going into hospital and being aware that everybody has the same condition, you then do become a lot more aware of some of the anorexia traits and you do sort of take them on” AW08

These women reported copying others and adopting their anorexic values as a way of camouflaging and passing in the neurotypical world.

Yet, although all of the women reported issues around their sense of self, for only a few of them this resulted in over-evaluation of their weight and shape, as outlined in the examples above. Most autistic women stressed that weight loss was not the initial aim of their ED behaviour, but rather a secondary and unintentional consequence.

“What I wanted was to be able to restrict food and over-exercise without losing weight. So that’s why it was so atypical. It was more like behaviours that I engaged with to feel calm, but would lead to catastrophic weight loss.” AW13

Assumptions by others that body image issues drove their ED behaviours made these women feel even more misunderstood and alienated, further contributing to their feelings of being different.

The role of weight and shape concerns was an area where some parents’ perceptions differed from those of autistic women. Several parents assumed that weight and shape concerns must be directly related to their daughters’ low sense of self and ED. However, a few also reported that body image issues did not play a role for their daughters' ED, or recognised the more nuanced reasons behind apparent body image issues and their relationship to the underlying autism, as described by the autistic women.

In line with the account that most autistic women’s ED is not primarily driven by the influence of weight and shape on self-evaluation, healthcare professionals noted that many autistic women seemed less drawn to comparing their appearance to others or taking pride in their weight loss. They also reported that autistic women tend to show less competitive behaviours in inpatient settings than other women with AN.

“When you unpick it, it’s not driven the same way, it’s not about body image, they couldn’t care less what other people think about their body.” HCP10

#### Emotional Difficulties

Many women reported that they had longstanding difficulties identifying, regulating, and communicating their emotions, resulting in emotional confusion and feelings of being overwhelmed. They also reported regularly having difficult and emotionally upsetting experiences, and some healthcare professionals suggested that autistic individuals might be particularly vulnerable to having traumatic and difficult experiences. Participants' accounts suggest that autistic women with AN may use restriction and other ED behaviours, such as exercise, in order to numb or distract themselves from overwhelming and confusing emotions.

This is something some reported to have discovered accidentally but then learnt to use purposefully. This woman’s quote illustrates how restriction offered a solution to her previously uncontrollable meltdowns:

“When I was restricting my eating, I would get this feeling of just calmness, and I know that I am safer, I know that I am not going to experience these meltdowns that made me feel embarrassed and frightened.[…] So I was no longer just losing it.” AW03

Healthcare professionals recognised how EDs in autistic women often relate to other mental health difficulties, particularly anxiety.

“[Their ED] is a way of channelling anxiety. They can just worry about food and nothing else and that feels more manageable than everything in their life that feels horrendous.” HCP09

During the interviews, almost all of the women described additional mental health difficulties, which they saw as being closely intertwined with their autism and their ED.

“My OCD [obsessive compulsive disorder] started to get worse as I started to fight my eating disorder. I just seem to have kind of variations on the same theme, with the OCD and with the eating disorder. The problem seemed to be not what the content of my thoughts was, but how I thought.” AW05.

Similarly, this woman’s quote illustrates the complex interplay between autism, difficulties with emotions, interoceptive difficulties and ED behaviours:

“I misinterpret [emotions] as physical symptoms and I get very anxious about it: Am I unwell? Am I going to vomit? And that’s when I stop eating because I know that will dampen things down and calm them, so my emotions are feeding into my eating disorder behaviours, whereas I think my difficulties in perhaps coping with emotions stem perhaps more from the autism.” AW08

Giving up on their ED behaviours, but lacking alternative ways of coping, was one of the greatest challenges in recovery:

“When she had a BMI of 12, she had that control because she had no hormones, no emotions, no nothing. Apart from the fact it might kill you, it was quite good for her. But once she was getting better, all those thoughts flooded back into her brain, and her mind was feeling an awful lot worse.” P03

“I sometimes imagine life without [AN], and then think, well actually, I would still have a lot of problems, but I wouldn’t have my coping mechanism.” AW05

#### Thinking Styles

Participants talked about several autistic thinking styles contributing to AN in autistic women, including literal thinking, obsessive and intense interests and rigid thinking, because they made them more vulnerable to develop rules around eating and food, and/or made it more difficult to shift their focus away from these rules once they were established. This was mentioned by all participants across groups, although the autism-related thinking styles that they suggested give rise to eating difficulties varied between individual participants. Many participants mentioned several thinking styles as being relevant:

“The autism and the routine and rigid thinking maintained the eating disorder. I think that’s why my recovery has taken so long for me to get to what I would call true recovery. For me, the anorexia is just a symptom, and the cause is the autism.” AW03

While participants acknowledged that these patterns of thinking became more entrenched with the persistence of the ED, they reported that they had pre-dated their ED and were closely linked to their autism.

“We know that some of those traits get more marked when they’re starved, and when they’re weight restored they improve. But for these women they never get completely better, which is one of the reasons to think they are enduring traits, rather than states, pre-dating the eating disorder.” HCP01.

##### Literal Thinking

In some cases, processing information in a literal way was thought to have led to distorted thinking around healthy eating and body image, which then gave rise to ED cognitions and behaviours. Parents in particular noticed that their daughters tended to make sense of the world in a very ‘black-and-white’ way.

“She takes things as absolutely true and cannot cope with nuances, untruths, or lack of clarity. This shades over into ‘all or nothing thinking’ too – “If I’m not thin then I’m fat and horrible”, with nothing in between.” P13

In many cases, overheard comments, public health advice, and lessons at school about healthy eating, such as “fat is bad for you”, were described as initially giving rise to rigid rules about eating and exercise and thus leading to the development of the ED.

##### Intense Interests

Obsessive thinking and intense ‘special’ interests related to ED behaviours also contributed to autistic women’s AN.

“She has always had obsessions with things, and once she had got on to healthy eating and food, that became extreme and made her very ill very quickly.” P11

For women in this study, such interests included exercise, nutrition, veganism or environmental concerns. A passion for counting and monitoring numbers, such as counting calories or looking for patterns in the numbers on weighing scales, was also common. For many autistic women these interests were described as an important source of enjoyment and a way to alleviate anxiety and bring calmness, which contributed to their persistence.

##### Rigid Thinking

Another autism-related thinking style that was thought to give rise to and maintain ED in autistic women was rigid and inflexible thinking. Participants described how autistic women’s rigid thinking resulted in difficulty with planning daily tasks and adapting to changing demands in day-to-day life, which in turn caused stress and emotional upset. Many participants felt that this rigid thinking style also made it harder to overcome anorexic thinking patterns and to not fall back into behaviours out of habit.

“Sometimes it’s just habit that I will [engage in ED behaviours], because that’s what I have done for the last 15 years, rather than a driven behaviour, if that makes sense.” AW04

However, some participants acknowledged that rigidity could also be an important tool for recovery, potentially driving the determination to get better.

“I think the ASD is making it so difficult to shift her thoughts.[…] I know that once she’s made up her mind about something, it’s very difficult to change it. So I live in hope that one day she’ll decide she’s going to get better, because if she does, because she’s so determined, she will do it. But until she makes that decision, it’s a battle.” P04.

#### Need for Control and Predictability

Participants described how autistic women’s rigid thinking and difficulty coping with change, which they saw as linked to their autism, elicits a need for control and predictability. Women seemed to address this need through controlling their food intake, sometimes in a ritualised fashion.

While most autistic women recalled that they could cope in early childhood when their life was more structured, they often started to struggle around the onset of puberty. Parents and healthcare professionals in particular felt that hormonal changes and resulting emotional extremes during this time further exaggerated feelings of confusion and perceived loss of control.

Stressful life events with unpredictable outcomes, such as illness or conflict in the family, or transitions to a new school or university, were also described as leading to worsening of eating behaviours. Although several women noticed these patterns, this was particularly clear to parents and healthcare professionals.

Being able to take control of something, having clear rules to follow and creating predictability were understood as powerful functions of AN in the autistic women. Autistic women’s inherent need for control, difficulty with change, and tendency to follow routines also made recovery difficult, and in some cases even made them doubt whether they could overcome their ED.

“I seem to have a strong need for control; I will always try and fill it with something. And if I could get rid of that, if I could learn to think differently… that would probably be the only way I could really recover.” AW03

### Autism-Specific Model of Restrictive Eating Difficulties

**Fig. 1:**
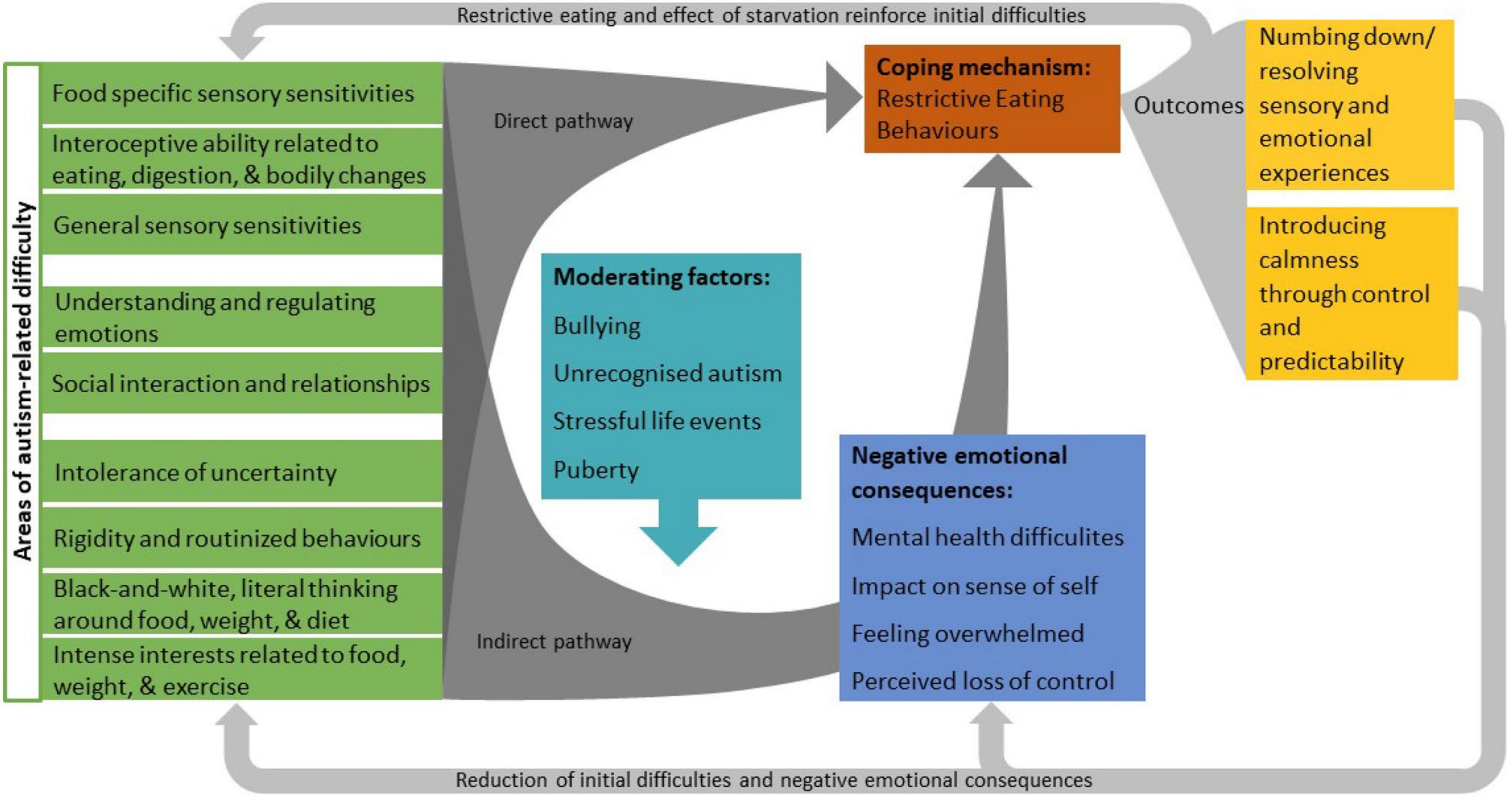
Proposed model of autism-specific mechanism underlying restrictive eating difficulties

Based on these findings, we developed a theoretical model based on hypothesised autism-specific mechanisms for restrictive eating difficulties (Fig. 1).

We propose that autism may give rise to restricted eating behaviours via a direct pathway and an indirect pathway. It seems that there are a range of autism-related difficulties that autistic individuals who develop restrictive eating difficulties might experience in their life. These difficulties seem to relate to core autistic traits, such as sensory sensitivities, social and emotional difficulties, and their cognitive profiles. In the direct pathway, autism-related difficulties, which revolve around food and ED-related behaviours, are suggested to increase risk of severe restrictive eating. For example, food-related sensory aversions or special interests focused on eating or exercise may contribute directly to restrictive eating and related behaviours. In the indirect pathway, autism-related difficulties are thought to give rise to negative emotional consequences, and we suggest that restrictive eating is used as a maladaptive attempt to cope with this. For example, for an undiagnosed and unsupported autistic individual, a longstanding history of social ostracism and peer victimisation can give rise to emotional distress; and they may discover that restricting food intake serves to numb these feelings, whilst the experience of gaining control over their calorie intake helps assuage anxiety. It is important to note that external factors, such as being bullied, being misunderstood because the individual’s autism is not recognised, stressful life events, or puberty, are likely to play an important role in the indirect pathway. These may moderate the relationship between autism-specific difficulties and emotional consequences. The nature of the initial difficulties and the combination of different factors experienced by an individual may result in a variety of restrictive eating presentations. For example, a strong aversion to food characteristics might result in a more “ARFID-type” presentation, whereas issues in social relationships or experiences, which affect the individuals sense of identity and direct their focus towards weight and shape, may result in a more traditional AN presentation. It is hypothesised that restrictive eating behaviours are maintained because their outcomes directly reduce the individual’s autism-related difficulties and their negative emotional consequences by: (1) numbing down or resolving some of the sensory and emotional experiences; (2) introducing calmness through giving a sense of control and providing predictability. At the same time, the ED and effect of starvation may work against this ameliorating effect and exacerbate some of the initial difficulties.

## Discussion

This qualitative study specifically investigates how AN develops and is maintained in autistic women from the combined perspectives of autistic women with AN, parents and healthcare professionals. Our interviews suggest that autistic women with AN experience their autism and AN as closely intertwined. AN in autistic individuals seems to relate to sensory sensitivities, difficulties with social interaction and relationships, autistic women’s sense of self and identity, difficulties with emotions, autistic thinking styles and a need for control and predictability. Further, we draw on these findings to propose a theoretical model of the hypothesised processes by which autistic traits may give rise to and maintain restrictive eating difficulties in autistic individuals.

Parents were able to contribute a developmental perspective and insights into areas of personal history that the autistic women had more difficulty reflecting upon, such as triggering factors that preceded episodes of disordered eating. Healthcare professionals were able to identify common patterns of behaviour from having worked with multiple autistic women, while also comparing them to their non-autistic clients. Although some clinicians might lack the confidence to treat these individuals (Kinnaird et al. 2017), it is notable that the healthcare professionals in our study demonstrated relevant clinical insight and discussed similar themes to the autistic women. Given parents and practitioners role in facilitating access to and providing support, greater awareness of different potential presentations of restrictive eating difficulties in autism and a shared understanding of a women’s difficulties seems to be vital for improving outcomes for affected girls and women.

The findings of the current study accord with those of Kinnaird et al. (2019b), even though they were conducted in separate samples, and the first authors of the current paper were not aware of their findings at the time of analysis. Both studies suggest that autistic women experienced their AN and autism to be deeply interlinked, with autism-related traits both contributing towards AN development and making recovery more challenging (Kinnaird et al. 2019b). This study added to Kinnaird et al.’s (2019b) finding by illuminating some of the underlying processes through which autism-related traits might introduce the individual to restrictive eating behaviours or maintain an ED once it has developed. Kinnaird et al. (2019b) suggested that many of the factors that were identified as contributing to the development and maintenance of AN, such as sensory sensitivity and social communication difficulties, also cause autistic women difficulty engaging in treatment.

Kinnaird et al. (2019b) reported that participants described how a desire to lose weight, low self-esteem, and body image issues were less relevant in the development of their illness compared to other motivations that are less commonly associated with AN. In line with this, many women in our study emphasised that weight and shape concerns were not driving their restrictive eating behaviours. Further, when weight and shape concerns did play a role, this study was able to add insights into autism-related motivations that seem to underpin such preoccupations. In contrast to Kinnaird et al.’s (2019b) findings, low self-esteem did emerge as highly relevant for women in the current sample, who felt different and struggled with their sense of self because of their autism not being recognised. However, this deviated from traditional understanding of low-self-esteem in EDs, as it was closely linked to these women being autistic.

Based on the findings from our interviews, we developed a theoretical model of autism-specific mechanisms for restrictive eating difficulties, hypothesising how autism-related difficulties may contribute to the development and maintenance of restrictive eating behaviours in autistic individuals (Fig. 1). Our model proposes that restrictive eating behaviours and consequent difficulties in autistic individuals can stem directly from their autism, for example reflecting sensory aversions to foods. Eating difficulties may also arise as an attempt to cope with the indirect challenges of being autistic, such as consequent mental health difficulties or issues around identity. Engaging in restrictive eating behaviours and the effect of starvation seem to numb or resolve emotional and sensory overload, and controlling food intake can counter anxiety arising from being in an unpredictable environment. Each of the themes, which were captured by thematic analysis (see Table 2), spans multiple elements of this model (Fig. 1). The model illustrates how the different themes may relate to and interact with each other, and thus emphasises potential processes and underlying mechanisms through which autism might give rise to and maintains restrictive eating behaviours, which in their extreme take on the form of an ED. The additional theoretical discussion of the themes and data, which was part of the model development, added another layer of interpretation, such as the conceptualisation that underlying factors might have different types of influence (i.e. direct and indirect) on restrictive eating difficulties or that causing and maintaining factors could be categorised as either autism-related difficulties, negative emotional consequences, or external influences.

Many elements of the proposed model have been established in both AN and autistic populations, which supports their relevance for AN in autism. For example, both autistic individuals and those with AN present with social difficulties (Zucker et al. 2007), emotional dysregulation (Oldershaw et al. 2015; Mazefsky 2015), high rates of intolerance of uncertainty (Brown et al. 2017; South and Rodgers 2017), rigid thinking (Coniglio et al. 2017; Westwood et al. 2016), and even general and food-specific sensory sensitivities (Crane et al. 2009; Tonacci et al. 2019; Zucker et al. 2013; Kinnaird et al. 2018). However, few studies have looked at these factors in relation to autism and AN within the same sample, and studies tend to use different forms of measurement, which makes direct comparison between populations difficult.

Our focus on autistic women with AN and the use of qualitative methodology meant that we do not know to what extent the proposed themes apply to women with AN who are not autistic, although healthcare professionals provided some insight by comparing their experience with both groups. Given the overlap between the two conditions (Westwood and Tchanturia 2017), it may be that some of the factors proposed by the current study are autism-specific, and that (unrecognised) autistic individuals are driving observations made in AN samples. It will be important for future research to assess whether there are indeed differences in these factors between women with AN with high levels of autistic traits and those without autism. Another possibility is that some proposed factors are relevant to both autistic and non-autistic women with AN. If this is the case, there might still be subtle yet clinically meaningful differences in terms of how these factors present. For example, other models of AN suggest that emotional difficulties in AN tend to relate to intolerance of negative emotions in the self and others, resulting in emotional avoidance (Treasure and Schmidt 2013; Mansour et al. 2016), whereas the autistic women in the current study seemed to have an underlying inability to identify and regulate emotions and struggled with consequent emotional confusion. Finally, some factors might be general risk factors for AN, but given their close association with autistic behaviours, they are likely to affect autistic women disproportionately, both in terms of severity and number.

Similarly, while some elements of the model, such as food-related sensory sensitivities, seem particularly relevant to restriction and disordered eating in autism, other components might also be relevant to other mental health conditions. This could explain the co-occurring mental health difficulties experienced by autistic women with AN in our sample, which is in line with high rates of additional mental health conditions reported in similar samples (e.g. Westwood et al. 2017b). Some elements of the model, such as intolerance of uncertainty or difficulties regulating emotions, have been associated with other mental health difficulties and maladaptive behaviours, such as addiction and substance abuse, in autistic individuals (van Wijngaarden-Cremers and van der Gaag 2015; South and Rodgers 2017; Mazefsky 2015). Thus, they may be shared vulnerability factors for poor mental health outcomes in autism.

The current study exclusively focused on females. Both AN and autism are considered to have somewhat gender-specific presentations (Stanford and Lemberg 2012; Hiller et al. 2014; Lai et al. 2015; Hull et al. 2017), which raises the possibility that interactions between autism and AN may be different in females and males. With the prevalence rates of AN being much higher in females than males (Bulik et al. 2006; Nagl et al. 2016), recruiting sufficient numbers of autistic males with AN to adequately capture their experience would have been beyond the scope of this project. The applicability of the proposed model to autistic males, non-binary and transgender people with restrictive eating difficulties warrants further investigation.

Even though this research focused on autistic women with AN, the findings highlight potential overlap with other restrictive EDs and seem to have relevance for restrictive eating difficulties in autistic individuals more generally. For most women in our sample, weight and shape concerns did not seem to be driving their ED, even though this is commonly assumed to be the case for individuals with AN (American Psychological Association 2013; Fairburn et al. 1999). Instead, their restrictive eating seemed to be driven by other factors, such as food-specific sensory sensitivities, a desire to avoid certain bodily sensations, or an absence of hunger signals. For some women there seemed to be behavioural parallels to individuals with ARFID. ARFID is a recently introduced diagnostic ED category, characterised by avoidant and restrictive eating associated with failure to meet nutritional and/or energy requirements leading to significant weight loss or failure to gain expected weight; nutritional deficiencies; and/or significant difficulties with psychosocial functioning, but without the body shape issues that typify AN (American Psychological Association 2013). Individuals with ARFID tend to restrict their food intake for reasons such as avoidance of sensory aspects of food, lack of interest in food, or because of feared negative consequences (Thomas et al. 2017), and autism commonly co-occurs with ARFID (Nicely et al. 2014). Even though ARFID can present across the lifespan, it is more commonly considered in children, and thus may be overlooked or misdiagnosed as AN in adult women with low weight (Becker et al. 2019). The model we developed purposefully refers to restrictive eating difficulties in general, rather than just AN, as our findings do not necessarily suggest that the proposed autism-specific mechanisms are limited to a specific diagnostic category and/or severity level. Future research should investigate the apparent lack of weight and shape concerns in autistic women with restrictive eating difficulties, and include women with a variety of restrictive eating presentations to establish whether elements of the proposed model also apply to autistic individuals with restrictive eating disorders other than AN and restrictive eating difficulties at a sub-clinical level.

All women in our sample received their autism diagnosis in adulthood (mean age = 29.4 years), often years after first receiving treatment for AN (mean age = 17.4 years). Both being female and having other co-occurring mental health conditions are risk factors for delayed or missed autism diagnosis, and living with undiagnosed autism is associated with the development of mental health difficulties (Brown et al. 2019; Bargiela et al. 2016; Leedham et al. 2019). It is unclear whether some of the factors identified in this study would have affected autistic women differently if their autism had been recognised and supported earlier in life. Early autism diagnosis and specialist intervention for autistic girls and women at risk of restrictive eating difficulties may help to prevent the development or worsening of ED symptoms. However, ED clinicians self-report lack of confidence in identifying autistic individuals in their care (Kinnaird et al. 2017), and existing screening and diagnostic tools, including the AQ-10 (Allison et al. 2012) used in this study, are poorly equipped to reliably detect autistic traits in ED samples (Westwood and Tchanturia 2017). Relying on the AQ-10 to confirm presence of high levels of autistic traits should therefore be noted as a limitation of the current study. Future research should work towards better identification of autistic traits in AN, which will benefit both clinical practice and research.

### Future Directions and Implications

This research suggests a variety of avenues for future research. For example, further qualitative work in other samples and using different approaches, such as grounded theory, could explore the applicability and refine the model proposed in this paper. Similarly, systematic clinical case studies could be used to confirm the relevance of the factors identified in this study and determine the role of other potential factors, including how women’s support networks (parents and professionals) might influence their ED experience. Longitudinal and group comparison studies could further establish the causal role of different factors and their relevance for individuals with different presentations.

This research has important implications for the treatment of autistic individuals in ED services and for preventing the development or worsening of restrictive eating difficulties in autistic individuals. The finding that autistic women with AN report causal and maintaining factors that are not traditionally associated with AN raises the possibility that autistic women with AN may have more enduring presentation and poorer outcomes (Nazar et al. 2018; Tchanturia et al. 2017) because standard treatments do not address autism-specific mechanisms underlying their EDs. There is a need for ED services to identify autistic individuals in their care and to adapt treatment accordingly. In the long-term, this research may also contribute to the development and testing of new autism-specific AN treatments. Preventative approaches should aim to support individuals at risk with their difficulties, particularly during meal times, and help them to develop alternative coping mechanisms. The theoretical model presented in this study was based on the thematic analysis of the insights of autistic women with AN, their parents and relevant HCPs. It therefore provides a useful initial framework for considering relevant issues affecting restricted eating in women with AN and autism or high levels of autistic traits. However, further work is needed to empirically test and refine the model proposed in this study to maximise its impact.

## Conclusion

In this study, we propose a theoretical model of the autism-specific mechanisms underlying restrictive eating difficulties based on the experiences of affected individuals and those involved in their care. Autistic women with AN experience their autism and ED as closely intertwined. Our findings suggest that AN in autistic women may be distinct from AN in non-autistic women in terms of its presentation and underlying mechanisms. Further research is required to test these novel insights about the nature of AN in autism. The findings of this study may directly benefit affected individuals by increasing awareness of autism-specific restrictive eating presentations, and helping ED services to improve the way they treat autistic individuals with AN.

## Electronic supplementary material

Below is the link to the electronic supplementary material.

Supplementary file1 (DOCX 33 kb)

## References

[CR1] Allison C, Auyeung B, Baron-Cohen S (2012). Toward brief "Red Flags" for autism screening: The Short Autism Spectrum Quotient and the Short Quantitative Checklist for Autism in toddlers in 1,000 cases and 3,000 controls [corrected]. Journal of the American Academy of Child and Adolescent Psychiatry.

[CR2] American Psychological Association (2013). Diagnostic and statistical manual of mental disorders.

[CR3] Autistica (2019). Discover - autism research network. Retrieved October 30, 2019, from https://www.autistica.org.uk/our-research/discover-network.

[CR4] Barbour RS (2001). Checklists for improving rigour in qualitative research: A case of the tail wagging the dog?. BMJ.

[CR5] Bargiela S, Steward R, Mandy W (2016). The experiences of late-diagnosed women with autism spectrum conditions: An investigation of the female autism phenotype. Journal of Autism and Developmental Disorders.

[CR6] Becker KR, Keshishian AC, Liebman RE, Coniglio KA, Wang SB, Franko DL (2019). Impact of expanded diagnostic criteria for avoidant/restrictive food intake disorder on clinical comparisons with anorexia nervosa. International Journal of Eating Disorders.

[CR7] Bentz M, Jepsen JR, Pedersen T, Bulik CM, Pedersen L, Pagsberg AK (2017). Impairment of social function in young females with recent-onset Anorexia Nervosa and recovered Individuals. Journal of Adolescent Health.

[CR8] Braun V, Clarke V (2006). Using thematic analysis in psychology. Qualitative Research in Psychology.

[CR9] Brown, C. M., Fuller-Tyszkiewicz, M., Krug, I., & Stokes, M. A. (2019). *Diagnostic overshadowing in autistic women.* Paper presented at the International Society for Autism Research annual Meeting, Montreal.

[CR10] Brown M, Robinson L, Campione GC, Wuensch K, Hildebrandt T, Micali N (2017). Intolerance of uncertainty in eating disorders: A systematic review and meta-analysis. European Eating Disorder Review.

[CR11] Bulik CM, Sullivan PF, Tozzi F, Furberg H, Lichtenstein P, Pedersen NL (2006). Prevalence, heritability, and prospective risk factors for anorexia nervosa. Archives of General Psychiatry.

[CR12] Carter N, Bryant-Lukosius D, DiCenso A, Blythe J, Neville AJ (2014). The use of triangulation in qualitative research. Oncology Nursing Forum.

[CR13] Cermak SA, Curtin C, Bandini LG (2010). Food selectivity and sensory sensitivity in children with autism spectrum disorders. Journal of the American Dietetic Association.

[CR14] Coniglio KA, Becker KR, Franko DL, Zayas LV, Plessow F, Eddy KT (2017). Won't stop or can't stop? Food restriction as a habitual behavior among individuals with anorexia nervosa or atypical anorexia nervosa. Eating Behaviors.

[CR15] Crane L, Goddard L, Pring L (2009). Sensory processing in adults with autism spectrum disorders. Autism.

[CR16] Fairburn CG, Shafran R, Cooper Z (1999). A cognitive behavioural theory of anorexia nervosa. Behaviour Research and Therapy.

[CR17] Fereday J, Muir-Cochrane E (2006). Demonstrating rigor using thematic analysis: A hybrid approach of inductive and deductive coding and theme development. International Journal of Qualitative Methods.

[CR18] Gideon N, Hawkes N, Mond J, Saunders R, Tchanturia K, Serpell L (2016). Development and psychometric validation of the EDE-QS, a 12 item short form of the Eating Disorder Examination Questionnaire (EDE-Q). PLoS ONE.

[CR19] Gillberg C (1983). Are autism and anorexia nervosa related?. British Journal of Psychiatry.

[CR20] Guest G, Bunce A, Johnson L (2016). How many interviews are enough?. Field Methods.

[CR21] Hill CE, Thompson BJ, Williams EN (1997). A guide to conducting consensual qualitative research. The Counseling Psychologist.

[CR22] Hiller R, Pellicano L (2013). Anorexia and autism—A cautionary note. The Psychologist.

[CR23] Hiller RM, Young RL, Weber N (2014). Sex differences in autism spectrum disorder based on DSM-5 criteria: Evidence from clinician and teacher reporting. Journal of Abnormal Child Psychology.

[CR24] Huke V, Turk J, Saeidi S, Kent A, Morgan JF (2013). Autism spectrum disorders in eating disorder populations: A systematic review. European Eating Disorder Review.

[CR25] Hull L, Mandy W, Petrides K (2017). Behavioural and cognitive sex/gender differences in autism spectrum condition and typically developing males and females. Autism.

[CR26] Kinnaird E, Norton C, Pimblett C, Stewart C, Tchanturia K (2019). Eating as an autistic adult: An exploratory qualitative study. PLoS ONE.

[CR27] Kinnaird E, Norton C, Stewart C, Tchanturia K (2019). Same behaviours, different reasons: What do patients with co-occurring anorexia and autism want from treatment?. International Review of Psychiatry.

[CR28] Kinnaird E, Norton C, Tchanturia K (2017). Clinicians' views on working with anorexia nervosa and autism spectrum disorder comorbidity: A qualitative study. BMC Psychiatry.

[CR29] Kinnaird E, Stewart C, Tchanturia K (2018). Taste sensitivity in anorexia nervosa: A systematic review. International Journal of Eating Disorders.

[CR30] Kuschner ES, Eisenberg IW, Orionzi B, Simmons WK, Kenworthy L, Martin A (2015). A preliminary study of self-reported food selectivity in adolescents and young adults with Autism Spectrum Disorder. Research in Autism Spectrum Disorders.

[CR31] Lai M-C, Lombardo MV, Auyeung B, Chakrabarti B, Baron-Cohen S (2015). Sex/gender differences and autism: Setting the scene for future research. Journal of the American Academy of Child and Adolescent Psychiatry.

[CR32] Lang K, Larsson EEC, Mavromara L, Simic M, Treasure J, Tchanturia K (2016). Diminished facial emotion expression and associated clinical characteristics in anorexia nervosa. Psychiatry Research.

[CR33] Leedham A, Thompson A, Smith R, Freeth M (2019). ‘I was exhausted trying to figure it out’: The experiences of females receiving an autism diagnosis in middle to late adulthood. Autism.

[CR34] Lever AG, Geurts HM (2016). Psychiatric co-occurring symptoms and disorders in young, middle-aged, and older adults with Autism Spectrum Disorder. Journal of Autism and Developmental Disorders.

[CR35] Loomes R, Hull L, Mandy WPL (2017). What is the male-to-female ratio in Autism Spectrum Disorder? A systematic review and meta-analysis. Journal of the American Academy of Child and Adolescent Psychiatry.

[CR36] Mansour S, Rozenblat V, Fuller-Tyszkiewicz M, Paganini C, Treasure J, Krug I (2016). Emotions mediate the relationship between autistic traits and disordered eating: A new autistic-emotional model for eating pathology. Psychiatry Research.

[CR37] Mason D, McConachie H, Garland D, Petrou A, Rodgers J, Parr JR (2018). Predictors of quality of life for autistic adults. Autism Research.

[CR38] Mays N, Pope C (2000). Assessing quality in qualitative research. BMJ.

[CR39] Mazefsky CA (2015). Emotion regulation and emotional distress in Autism Spectrum Disorder: Foundations and considerations for future research. Journal of Autism and Developmental Disorders.

[CR40] Nagl M, Jacobi C, Paul M, Beesdo-Baum K, Hofler M, Lieb R (2016). Prevalence, incidence, and natural course of anorexia and bulimia nervosa among adolescents and young adults. European Child and Adolescent Psychiatry.

[CR41] Nazar BP, Peynenburg V, Rhind C, Hibbs R, Schmidt U, Gowers S (2018). An examination of the clinical outcomes of adolescents and young adults with broad autism spectrum traits and autism spectrum disorder and anorexia nervosa: A multi centre study. International Journal of Eating Disorders.

[CR42] Nicely TA, Lane-Loney S, Masciulli E, Hollenbeak CS, Ornstein RM (2014). Prevalence and characteristics of avoidant/restrictive food intake disorder in a cohort of young patients in day treatment for eating disorders. Journal of Eating Disorders.

[CR43] Nielsen S, Anckarsater H, Gillberg C, Gillberg C, Rastam M, Wentz E (2015). Effects of autism spectrum disorders on outcome in teenage-onset anorexia nervosa evaluated by the Morgan-Russell outcome assessment schedule: a controlled community-based study. Molecular Autism.

[CR44] Oldershaw A, Lavender T, Sallis H, Stahl D, Schmidt U (2015). Emotion generation and regulation in anorexia nervosa: A systematic review and meta-analysis of self-report data. Clinical Psychology Review.

[CR45] Oldershaw A, Treasure J, Hambrook D, Tchanturia K, Schmidt U (2011). Is anorexia nervosa a version of autism spectrum disorders?. European Eating Disorders Review.

[CR46] Pistrang N, Barker C (2012). Varieties of qualitative research: A pragmatic apprach to selecting methods. APA handbook of reserach methods in psychology.

[CR47] Pope C, Ziebland S, Mays N (2000). Analysing qualitative data. BMJ.

[CR48] NVivo. (2018). Qualitative data analysis software. (Vol. Version 12). Melbourne, Australia: QSR International Pty Ltd.

[CR49] Simonoff E, Pickles A, Charman T, Chandler S, Loucas T, Baird G (2008). Psychiatric disorders in children with autism spectrum disorders: prevalence, comorbidity, and associated factors in a population-derived sample. Journal of the American Academy of Child and Adolescent Psychiatry.

[CR50] South M, Rodgers J (2017). Sensory, emotional and cognitive contributions to anxiety in Autism Spectrum Disorders. Frontiers in Human Neuroscience.

[CR51] Stanford SC, Lemberg R (2012). A clinical comparison of men and women on the Eating Disorder Inventory-3 (EDI-3) and the Eating Disorder Assessment for Men (EDAM). Eating Disorders.

[CR52] Tchanturia K, Adamson J, Leppanen J, Westwood H (2017). Characteristics of autism spectrum disorder in anorexia nervosa: A naturalistic study in an inpatient treatment programme. Autism.

[CR53] Tchanturia K, Larsson E, Adamson J (2016). How anorexia nervosa patients with high and low autistic traits respond to group Cognitive Remediation Therapy. BMC Psychiatry.

[CR54] Tchanturia K, Smith E, Weineck F, Fidanboylu E, Kern N, Treasure J (2013). Exploring autistic traits in anorexia: A clinical study. Molecular Autism.

[CR55] Thomas J, Lawson E, Micali N, Misra M, Deckersbach T, Eddy K (2017). Avoidant/Restrictive Food Intake Disorder: A three-dimensional model of neurobiology with implications for etiology and treatment. Current Psychiatry Reports.

[CR56] Tonacci A, Calderoni S, Billeci L, Maestro S, Fantozzi P, Ciuccoli F (2019). Autistic traits impact on olfactory processing in adolescent girls with Anorexia Nervosa restricting type. Psychiatry Research.

[CR57] Treasure J (2013). Coherence and other autistic spectrum traits and eating disorders: building from mechanism to treatment. The Birgit Olsson lecture. Nord Journal of Psychiatry.

[CR58] Treasure J, Schmidt U (2013). The cognitive-interpersonal maintenance model of anorexia nervosa revisited: A summary of the evidence for cognitive, socio-emotional and interpersonal predisposing and perpetuating factors. Journal of Eating Disorders.

[CR59] Vagni D, Moscone D, Travaglione S, Cotugno A (2016). Using the Ritvo Autism Asperger Diagnostic Scale-Revised (RAADS-R) disentangle the heterogeneity of autistic traits in an Italian eating disorder population. Research in Autism Spectrum Disorders.

[CR60] van Wijngaarden-Cremers PJM, van der Gaag RJ, Dom G, Moggi F (2015). Addiction and autism spectrum disorder. Co-occurring addictive and psychiatric disorders: A practice-based handbook from a European perspective.

[CR61] Westwood H, Mandy W, Simic M, Tchanturia K (2018). Assessing ASD in adolescent females with Anorexia Nervosa using clinical and developmental measures: A preliminary investigation. Journal of Abnormal Child Psychology.

[CR62] Westwood H, Mandy W, Tchanturia K (2017). The association between symptoms of autism and neuropsychological performance in females with Anorexia Nervosa. Psychiatry Research.

[CR63] Westwood H, Mandy W, Tchanturia K (2017). Clinical evaluation of autistic symptoms in women with anorexia nervosa. Molecular Autism.

[CR64] Westwood H, Stahl D, Mandy W, Tchanturia K (2016). The set-shifting profiles of anorexia nervosa and autism spectrum disorder using the Wisconsin Card Sorting Test: A systematic review and meta-analysis. Psychological Medicine.

[CR65] Westwood H, Tchanturia K (2017). Autism spectrum disorder in anorexia nervosa: An updated literature review. Current Psychiatry Reports.

[CR66] Zucker NL, Losh M, Bulik CM, LaBar KS, Piven J, Pelphrey KA (2007). Anorexia nervosa and autism spectrum disorders: Guided investigation of social cognitive endophenotypes. Psychological Bulletin.

[CR67] Zucker NL, Merwin RM, Bulik CM, Moskovich A, Wildes J, Groh J (2013). Subjective experience of sensation in Anorexia Nervosa. Behaviour Research and Therapy.

