# Supplementary material for: “For Me, the Anorexia is Just a Symptom, and the Cause is the Autism”: Investigating Restrictive Eating Disorders in Autistic Women
Source: J Autism Dev Disord. 2020 Apr 9;50(12):4280–96. doi: 10.1007/s10803-020-04479-3 (PMC7677288; doi:10.1007/s10803-020-04479-3)
Supplement: Supplementary file 1 — Supplementary file1 (DOCX 33 kb) [file 10803_2020_4479_MOESM1_ESM.docx]

**Interview Schedule for Autistic Women**

Hello, thank you so much for agreeing to take part in our study, we really appreciate it.

- That there are no right or wrong answers, we are just interested in your views and experience.
- Please let me know if you would like me to repeat any questions or ask it in a different or more specific way.
- It is important for you to know that you don’t need to answer any questions that you don't feel comfortable with, we can skip them, or go back to them later.
- Don’t worry if you feel like you have forgotten to include anything. At the end I will check with you if you would like to add anything and if there are any topics you think we have missed that you might like to tell me about.

*Note for interviewer:*

*General prompts that can be used throughout the interview*

- *What/why/who/how?*
- *Can you give me an example, or describe a situation where this was the case?*
- *Could you talk me through this experience/situations?*

*Option: use written guide to interview topics to support the interview process.*

1. Experience and diagnosis of autism

To start off, could you tell me about your autism?

- What is autism like for you in your day to day life?
  - Are there ways in which autism makes your life more difficult?
  - Are there things that you’re better at than others?
  - When did you first notice ____?
- Has this changed with time/as you have grown up?
- What was it like to receive your autism diagnosis?
  - When were you diagnosed with autism?
  - Could you describe how you received your diagnosis? *(reason and process, e.g. school initiated assessment, seeking referral from GP, referral from other MH services)*
- What did you think of the diagnosis?
  - Did you think/suspect you might be on the autism spectrum prior to receiving a diagnosis, or was this something new to you?
  - How did you react when you received the diagnosis?
  - How did other people around you react when you received the diagnosis? (Think about family, friends, teachers/employers reactions)
- What, if anything, changed as a result of being diagnosed?

1. Experience and diagnosis of eating disorder

Could you to tell me about your own experiences of eating disorders.

- What was it like to receive your eating disorder diagnosis?
  - When did you receive an eating disorder diagnosis?
  - Could you describe the process of receiving your diagnosis? *(reason and process, e.g. seeking referral from GP, referral from other MH services)*
  - Was this before or after you received the autism diagnosis?
  - How did you react when you received your eating disorder diagnosis?
- Was there anything unusual with regard to your eating before this?
  - What was your eating like when you were younger?
- When did you notice your eating had become an issue /did someone else point it out to you?

1. Causes and maintaining factors

We know that eating disorders are complex and it can be difficult to understand why an eating disorder develops, for both professionals and the individual. But, do you have any insights as to why your eating disorder developed?

- - How did it start?
  - Are there any triggers for you?
  - Do you/did you value your eating disorder at any point?
- Is/Was your relationship to eating always difficult regardless of what else is/was going on in your life or are/were there particular times that makes/made eating easier/more difficult for you?

*If participant struggles to identify causes/maintaining factors:*

- In eating disorders research, there are many potential reasons why someone might engage in disordered eating. The reasons might apply to some people but not to others. These may or may not have impact you.
  - Some people feel a need to be in control;
  - Some don’t realise when they’re hungry and what/how much they should be eating;
  - Some get preoccupied with counting calories;
  - Some have difficulties with the smell or texture of different foods;
  - Some engage in restricted eating behaviours to connect and fit in with other people;
  - Some feel pressured by friends, society or things in the media;
  - Some are not happy with the way they look in the mirror;
  - Others want to lose weight
- Did any of these influence you?
- What other things might have played a role for your eating disorder?

*Offer an optional break*

1. Eating disorder services

Now I’m going to ask you some questions about your experiences with eating disorder services.

- Have you had any involvement with eating disorder services?
- Are you currently involved with them?
- Could you tell me about your experience in eating disorder services?

1. Referral/seeking help process

- What was the referral process like for you?
  - How were you referred to eating disorder services? (e.g. GP, other mental health services)
  - Who initiated your referral? Did you agree? How did you feel about being referred?
  - What was this process like for you? (Wait times?)

1. Experience of treatment

Once seen by a specialist, what treatment options were you offered?

- How did they try to help you?
- Did you take up this option? Why (not)?
  - What is/was it like for you?
  - Can you think of anything that seems to have been particularly helpful?
  - Can you think of anything that might have got in the way, or was difficult?
- What is/was your experience like with therapist/other staff?
- Are/were any other people involved? (Other patients encountered during treatment/family therapy?)

→ Was this consistent or was there anything that made [any of these things] easier/more difficult? Were there any circumstances that made it better/worse?

*If applicable discuss experience in inpatient/day patient treatment, individual therapy, group therapy.*

- - *Inpatient/day patient: What was the environment like on the ward, mealtime?*
  - *Individual or group therapy: Do you remember what kind of treatment you were offered? (e.g. CBT); What topics did the therapy cover? Were there any particular topics that were more/less helpful than others?*

1. Discharge/service experience outside of ED

*If you have been discharged,* what was this like?

- How did you feel about being discharged? (Positive aspects/worries?)
- Have you had any engagement with services since?
- Have you received input related to autism or eating from any other services than discussed already? Any other MH input?
- What other coping strategies or support (by professionals/other people in your life) has helped you/ do you find helpful?
- What one thing stood out to you as the most helpful so far?
- What could services have done differently?

1. Status Quo

How do you manage your eating now? How do you feel about your eating now?

1. Relationship between autism and eating disorder

- What role (if any), do you think, does/did your autism play in your experience of eating disorder?
- Are any other things you have noticed that might be different about the experience of eating disorders for autistic people?
  - Have you met anyone else with an eating disorder who doesn’t have autism? Did you notice any difference between your struggles with eating and food compared to them?

1. Summary

- Before we wrap up, is there anything else you would like to add? Anything we have missed? Any questions?

Thank you so much for your time. We’ll go through a quick debrief now before we finish.

**Interview Schedule for Family Members of Autistic Women**

Hello, thank you so much for agreeing to take part in our study, we really appreciate it.

- There are no right or wrong answers, we are just interested in your views and experience.
- Please let me know if you would like me to repeat any questions or ask it in a different or more specific way.
- It is important for you to know that you don’t need to answer any questions that you don't feel comfortable with, we can skip them, or go back to them later.
- Don’t worry if you feel like you have forgotten to include anything. At the end I will check with you if you would like to add anything and if there are any topics you think we have missed that you might like to tell me about.

*Note for interviewer: General prompts that can be used throughout the interview*

- *What/why/who/how?*
- *Can you give me an example, or describe a situation where this was the case?*
- *Could you talk me through this experience/situations?*

*Option: use written guide to interview topics to support the interview process.*

1. Introduction

To start off, could you tell me a bit about [your autistic family member with experience of eating disorder] and yourself.

- What is she like? Age? How does she spend her time? What are her interests?
- What is your relationship?
  - Do you live together?
  - Are there any other family members/important people?

2. Experience and diagnosis of autism

Could you tell me a bit about [your daughter's] autism?

- What is autism like for [her] in her day to day life?
  - Are there any things [she] finds particularly challenging?
  - Are there things [she] is good at? Or that are less of a problem for [her]?
  - When did you first notice these things?
- Has this changed over time/as she grew up?
- What was it like for her to receive an autism diagnosis?
- When was [she] diagnosed?
- Could you describe how [she] received her diagnosis? (*reason and process, e.g. seeking referral from GP, referral from other MH services*)
- What did you both think of the diagnosis?
  - Did you think/suspect she might be on the autism spectrum prior to her receiving a diagnosis, or was this something new to you?
  - How did both of you react when [she] received the diagnosis?
  - How did others around her react (family, friends, teachers/employers reactions)?
  - What, if anything, changed as a result of [her] being diagnosed?

3. Experience and diagnosis of eating disorder

Could you to tell me about [your daughter’s] experiences of eating disorders.

- What was it like for her to receive an eating disorder diagnosis?
- When did she receive an eating disorder diagnosis?
- Could you describe the process of her receiving the diagnosis? How was [she] diagnosed? (e.g. seeking referral from GP, referral from other MH services)
- Was this before or after the autism diagnosis?
- How did you react when she received her eating disorder diagnosis?
- Was there anything unusual with regard to her eating before this?
- What was her eating like when she was younger?
- When did you notice her eating had become an issue?

1. Causes and maintaining factors

We know that eating disorders are complex and it can be difficult to understand why an eating disorder develops, for professionals, the individual and family alike. But, Is there anything you think might have led up to her eating disorder?

- - How did it start?
  - Are there any triggers for [her]?
  - Can you see any way in which the eating disorder might have added value to her life at any point/ was a good thing?
- Is/Was her relationship to eating always difficult regardless of what else is/was going on in her life or did you notice that there are/were there particular times that makes/made eating easier/more difficult for her?

In eating disorders research, there are many potential reasons why someone might engage in disordered eating. The reasons might apply to some people but not to others.

- Some people feel a need to be in control;
- Some don’t realise when they’re hungry and what/how much they should be eating;
- Some get preoccupied with counting calories;
- Some have difficulties with the smell or texture of different foods;
- Some engage in restricted eating behaviours to connect and fit in with other people;
- Some feel pressured by friends, society or things in the media;
- Some are not happy with the way they look in the mirror;
- Others want to lose weight

Do you think any of these have influenced your daughter?

What other things might have played a role for her eating disorder?

*Offer an optional break*

5. Eating disorder services

Now I’m going to ask you some questions about [your daughter’s] experience in eating disorder services.

- Has she had any involvement with eating disorder services?
- Is she currently involved with them?
- In general, what do you think was/is her experience of eating disorder services?
- What is/was it like for her?
- What was it like for you (from a family member’s perspective)?

6. Referral/seeking help process

What was the referral process like?

- How was she referred to eating disorder services? (e.g. GP, other mental health services)
- Who initiated the referral?
- What was this process like for her? (Wait times?)

7. Experience of treatment

Once seen by a specialist, what treatment options were offered to her?

- How did they try to help?
- Did she take up these options? Why (not)?
- What is/was treatment like for her?
- Can you think of anything that seems to have been particularly helpful?
- Can you think of anything that might have got in the way, or was difficult for her?
- Were you involved in the treatment process at all? (e.g. family therapy)
- What is/was her relationship to her therapist/other staff?
- Are/were any other people (patients) involved? Do you think they (have) affect(ed) her in any way? In what way? Why not?
- Were there any circumstances that made it better/worse?

*If applicable discuss experience in inpatient/day patient treatment, individual therapy, group therapy.*

- - *Inpatient/day patient: What was the environment like on the ward, mealtime?*
  - *Individual or group therapy: Do you remember what kind of treatment your daughter was offered? (e.g. CBT); Are you aware of what topics her therapy covered? Were there any topics that were more/less helpful than others for her?*

8. Discharge/service experience outside of ED

*If she have been discharged,* what was this like?

- How did you feel about her being discharged? (Positive aspects/worries?)
- Has she had any engagement from services since?
- Has your daughter received input from any other services that related to her autism or eating? Any other MH input?
- What other coping strategies or support (by professionals/other people in her life) have helped her?
- What one thing stood out to you as the most helpful thing so far?
- What one thing should services have done differently?

9. Status Quo

- How are things now, in your opinion?

- How does she currently manage her eating?

10. Relationship between autism and eating disorder

- What role, do you think, does/did her autism play for her eating disorder?

- Are any other things you have noticed that might be different about the experience of eating disorders for autistic women?

- Do you know of anyone else with an eating disorder who doesn’t have autism? Did you notice any difference between [your daughter’s] struggles with eating and food compared to their difficulties?

11. Summary

- Before we wrap up, is there anything else you would like to add? Anything we have missed?

- Do you have any questions?

Thank you so much for your time. We’ll go through a quick debrief now before we finish.

**Interview Schedule for Eating Disorder Healthcare Professionals**

Hello, thank you so much for agreeing to take part in our study, we really appreciate it. We are looking to understand more about eating disorders, particularly anorexia nervosa, within autistic women and their experiences of eating disorders services, and we’d like your perspective on this topic. Please let me know if you would like me to repeat my question or ask it in a different way.  It is important for you to know that you don’t need to answer any questions that you don't feel comfortable with and that there are no right or wrong answers, only your answers. At the end I will check with you if you would like to add anything and if there are any topics you think we have missed that you might like to tell me about.

*General prompts:*

- *Can you expand on this point?*
- *Can you say a bit more about this?*
- *Can you give me an example?*
- *Could you talk me through this experience?*

1. Introduction:

What is your current job role?

How long have you worked in this role?

How long have you worked in a specialist eating disorder setting?

1. Professional experience and knowledge of eating disorders

To start off, could you tell me about your experience of anorexia nervosa within your profession?

- What's the context in which you work with females with anorexia (e.g. on the ward, in therapy)
- How would you describe the presentation of anorexia symptomatology in females in your work to someone who is not familiar with your field of work?
- Have you come across any difficulties with screening or carrying out diagnostic assessments with females with anorexia? Examples?

1. Professional experience and knowledge of autism

I’d like you to tell me about your experience of autism within your profession?

How familiar are you with autism, particularly in females?

- What is your understanding of autistic traits? Would you know what traits to look out for?
- In which ways do you think autism is/might be relevant in the work you do?
- Do you screen for or carry out diagnostic assessments for autism in females with anorexia? If so, in what situations? What are the challenges with this?

1. Relationship between autism and eating disorders

What are your thoughts/understanding of the relationship between autism and eating disorders?

- Have you ever come across it within your clinical practice? In what ways?
- Were there any challenges? If so, what were they?
- How might autistic women with anorexia differ from non-autistic women with anorexia?

We know that patients with anorexia can present with social communication difficulties, rigid behaviours and interests and might have difficulties to identify and cope with emotions.

- Would you consider these patients to be on the autism spectrum?
- Under what conditions would you consider these traits to be autistic in nature?
- Under what conditions would you consider these traits to be a consequence of their anorexia?

1. Maintaining factors of AN in autism

Based on your professional knowledge and/or experience, can you think of any potential contributing factors to the development of anorexia in autistic women?

- What factors might make them more or less likely to develop an eating disorder?
- What might be the main struggles for autistic women with anorexia?
- Can you think of certain situations that might make autistic women struggle more with their eating disorder? Or situations that make it better?

**Eating Disorders Services for Autistic Women**

1. Routes of referral

If you suspected a woman with anorexia might have autism, what would you/your service do?

- Who would you consult? Why?
- Would you investigate it yourself/within your service? If so, in which particular situations?
- Would you refer them to a specialist? If so, in which particular situations?

What challenges do you think an autistic woman might face when being referred for an eating disorder?

- Why these challenges in particular?

1. Treatment of eating disorders for autistic women

What challenges do you think autistic women might face when engaging with eating disorder services? How might autistic traits be challenging for patients with anorexia in a service context?

- In what ways might group therapy be a challenge for autistic women?
- (Engagement with staff/therapist/other patients; communication difficulties; disrupted routines; sensory/environmental concerns)
- In what ways might individual therapy be a challenge for autistic women?
- (Engagement with staff/therapist/other patients; communication difficulties; disrupted routines; environmental concerns)
- In what ways might inpatient or day patient treatment be a challenge for autistic women?
- (Engagement with staff/therapist/other patients; communication difficulties; disrupted routines; environmental concerns)

Do you think the care and needs of autistic women are different to the care and needs of a non-autistic women within eating disorders services?

- In what ways would they differ and why?
- What additional support might autistic women with anorexia require?

What might be some of the challenges that therapists and other staff members face when treating autistic women with anorexia?

What might be some of the challenges on a service level?

Could you suggest any therapeutic interventions or particular topics that might help an autistic woman with anorexia? (e.g. emotion regulation, therapeutic interventions involving central coherence/set shifting)

8. Summary

Before we wrap up, is there anything else you would like to add? Anything we have missed?

Do you have any questions?

Thank you so much for your time. We’ll go through a quick debrief now before we finish.

**Interview Schedule for Autism Healthcare Professionals**

Hello, thank you so much for agreeing to take part in our study, we really appreciate it. We are looking to understand more about eating disorders, particularly anorexia nervosa, within autistic women and their experiences of eating disorders services, and we’d like your perspective on this topic. Please let me know if you would like me to repeat my question or ask it in a different way.  It is important for you to know that you don’t need to answer any questions that you don't feel comfortable with and that there are no right or wrong answers, only your answers. At the end I will check with you if you would like to add anything and if there are any topics you think we have missed that you might like to tell me about.

*General prompts:*

- *Can you expand on this point?*
- *Can you say a bit more about this?*
- *Can you give me an example?*
- *Could you talk me through this experience?*

1. Introduction:

What is your current job role?

How long have you worked in this role?

How long have you worked in a specialist autism setting?

1. Professional experience and knowledge of autism

- To start off, could you tell me about your experience of autism in females within your profession?
- In what capacity might you come across autistic females in your work? (e.g. diagnostic, care coordination, therapy, consulting professionals from other services)
- In what ways might females with autism present differently to the traditional stereotypes of autistic presentation?
- How would you describe the presentation of autistic traits in females to someone who is not familiar with your field of work?
- Have you come across any difficulties with screening or carrying out diagnostic assessments with autistic females? Examples?

1. Professional experience and knowledge of eating disorders

I’d like you to tell me about your experience of eating disorders within your profession.

- How familiar are you with eating disorders, particularly anorexia? What's your understanding of eating disorder symptomatology? (Would you know what symptoms to look out for?)
- In which ways do you think anorexia might be/is relevant in the your work in autism?

1. Relationship between autism and eating disorders

What are your thoughts on the relationship between autism and eating disorders?

- Have you ever come across it within your clinical practice? In what ways?
- Were there any challenges? What were they?
- How might autistic women with anorexia differ from other autistic women who don’t have anorexia?
- Are there any other things **you** have noticed that might be different about the experience of eating disorders for autistic people?

1. Maintaining factors of AN in autism

Based on your professional knowledge and/or experience, can you think of any potential contributing factors to the development of anorexia in autistic women?

- What factors might make them more or less likely to develop an eating disorder?
- What might be the main struggles for autistic women with AN?
- Can you think of certain situations that might make autistic women struggle more with their eating disorder? Or make it better?

**Eating Disorders Services for Autistic Women**

1. Routes of referral

If you suspected an autistic woman might have an eating disorder, what would you do?

- Who would you consult? Why?
- Would you investigate it yourself and/or support them within your service? If so, why/in which particular situations?
- Would you refer them to a specialist? If so, why/in which particular situations?
- Do you screen for or carry out diagnostic assessments for anorexia/eating disorders in autistic females? In what situations? What are the challenges with this?

What challenges do you think autistic women in particular might face when being referred for an eating disorder?

1. Treatment of eating disorders for autistic women

What challenges do you think autistic women might face when engaging with eating disorder services?

- In what ways might group therapy be a challenge for autistic women?
- (Engagement with staff/therapist/other patients; communication difficulties; disrupted routines; environmental concerns)
- In what ways might individual therapy be a challenge for autistic women?
- (Engagement with staff/therapist/other patients; communication difficulties; disrupted routines; environmental concerns)
- In what ways might inpatient or day patient treatment be a challenge for autistic women?
- (Engagement with staff/therapist/other patients; communication difficulties; disrupted routines; environmental concerns)

Do you think the care and needs of an autistic women would be different to the care and needs of a non-autistic women within eating disorders services?

- In what ways would they differ and why?
- What additional support might an autistic women with anorexia require?

What might be some of the challenges that therapists and other staff members face when treating autistic women with anorexia?

- What might be some of the challenges on a service level?

Could you suggest any therapeutic interventions or particular topics that might help an autistic woman with anorexia? (e.g. emotion regulation, therapeutic interventions involving cognitive styles)

8. Summary

Before we wrap up, is there anything else you would like to add? Anything we have missed?

Do you have any questions?

Thank you so much for your time. We’ll go through a quick debrief now before we finish.
